# Urinary Excretion of Tetrodotoxin Modeled in a Porcine Renal Proximal Tubule Epithelial Cell Line, LLC-PK_1_

**DOI:** 10.3390/md15070225

**Published:** 2017-07-17

**Authors:** Takuya Matsumoto, Yui Ishizaki, Keika Mochizuki, Mitsuru Aoyagi, Yoshiharu Mitoma, Shoichiro Ishizaki, Yuji Nagashima

**Affiliations:** 1 Department of Environmental Sciences, Faculty of Life and Environmental Science, Prefectural University of Hiroshima, Shobara, Hiroshima 727-0023, Japan; yuii0q24@gmail.com (Y.I.); q623024rf@ed.pu-hiroshima.ac.jp (K.M.); aoyagi@pu-hiroshima.ac.jp (M.A.); mitomay@pu-hiroshima.ac.jp (Y.M.); 2 The Graduate School of Marine Science and Technology, Tokyo University of Marine Science and Technology, Minato, Tokyo 108-8477, Japan; ishizak@kaiyodai.ac.jp (S.I.); yujicd@kaiyodai.ac.jp (Y.N.)

**Keywords:** tetrodotoxin, urinary excretion, food poisoning, LLC-PK_1_, transcellular transport, organic cation transporter, organic cation/carnitine transporter, organic anion transporter, multidrug resistance-associated protein

## Abstract

This study examined the urinary excretion of tetrodotoxin (TTX) modeled in a porcine renal proximal tubule epithelial cell line, LLC-PK_1_. Time course profiles of TTX excretion and reabsorption across the cell monolayers at 37 °C showed that the amount of TTX transported increased linearly for 60 min. However, at 4 °C, the amount of TTX transported was approximately 20% of the value at 37 °C. These results indicate that TTX transport is both a transcellular and carrier-mediated process. Using a transport inhibition assay in which cell monolayers were incubated with 50 µM TTX and 5 mM of a transport inhibitor at 37 °C for 30 min, urinary excretion was significantly reduced by probenecid, tetraethylammonium (TEA), l-carnitine, and cimetidine, slightly reduced by *p*-aminohippuric acid (PAH), and unaffected by 1-methyl-4-phenylpyridinium (MPP+), oxaliplatin, and cefalexin. Renal reabsorption was significantly reduced by PAH, but was unaffected by probenecid, TEA and l-carnitine. These findings indicate that TTX is primarily excreted by organic cation transporters (OCTs) and organic cation/carnitine transporters (OCTNs), partially transported by organic anion transporters (OATs) and multidrug resistance-associated proteins (MRPs), and negligibly transported by multidrug and toxic compound extrusion transporters (MATEs).

## 1. Introduction

Tetrodotoxin (TTX) is a powerful neurotoxin that binds to voltage-gated sodium channels with very high affinity [1,2]. TTX is found in a variety of organisms, including pufferfish, goby, Astropectinidae (starfish), newts, poison dart frogs, blue-ringed octopuses, trumpet shells, Xanthid crabs, and horseshoe crabs [3]. TTX poisoning develops after the ingestion of TTX-containing organisms (e.g., pufferfish). We know that humans have eaten pufferfish since antiquity, because pufferfish bones have been unearthed from kitchen middens that date back to the Stone Age [4,5,6]. TTX poisonings have been reported in Japan, as well as in Australia, Bangladesh, Brazil, China, Singapore, Spain, Taiwan, Thailand, and the United States [7,8,9,10,11,12,13,14,15]. The symptoms of TTX poisoning depend on the amount of toxin ingested. While the minimum lethal dose of TTX is estimated to be 1–2 mg, it can be affected by hunger, satiety, drinking, etc., and the minimum toxic dose is unknown [16]. The initial sensory symptoms include a sense of numbness around the lips, tongue, and the most distal segments of the limbs. Mild cases present with sensory symptoms that are occasionally accompanied by gastrointestinal symptoms, such as nausea and vomiting. Moderate cases present with weakness of the distal and facial muscles, which induce bulbar palsy followed by ataxia and a lack of coordination. Severe cases present with systemic flaccid paralysis, respiratory failure, aphonia, and mydriasis. The most serious cases present with cardiovascular symptoms, such as hypotension, bradycardia, arrhythmias, and coma, in addition to respiratory failure [17,18].

Previous studies have found that plasma concentrations of TTX decrease rapidly, and are undetectable by 12–24 h after ingestion, although TTX can be detected in the urine four days after ingestion [19,20,21,22]. These findings indicate that, while TTX is primarily excreted into the urine, the mechanisms involved are not well understood. In the present study, we examined the urinary excretion of TTX modeled using a porcine renal proximal tubule epithelial cell line (LLC-PK_1_ cell monolayers) and investigated the effects of several transport inhibitors on the movement of TTX across these monolayers.

## 2. Results

**Figure 1:**
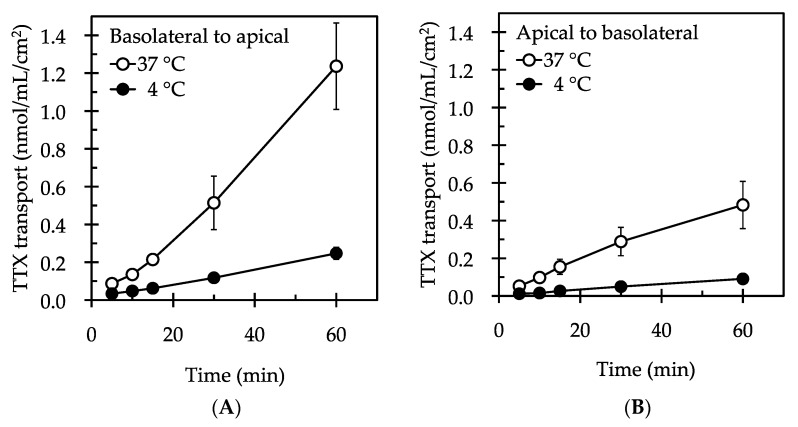
Time course profiles of TTX excretion (basolateral to apical) (**A**) and reabsorption (apical to basolateral) (**B**) across the LLC-PK_1_ cell monolayers. The cell monolayers were incubated at 37 °C or at 4 °C for up to 60 min with 50 µM TTX added to the basolateral (**A**) or apical side (**B**). Values are the mean ± SE of six different experiments performed in triplicate.

Time course profiles of TTX urinary excretion (from the basolateral to the apical side of the LLC-PK_1_ cell monolayers) are shown in Figure 1A. At an incubation temperature of 37 °C, the amount of TTX transported was 0.087 ± 0.015 nmol/mL/cm^2^ 5 min after incubation, which significantly increased to 1.237 ± 0.229 nmol/mL/cm^2^ 60 min after incubation (*p* < 0.05). When the incubation temperature was 4 °C, the amount of TTX transported was 0.034 ± 0.008 nmol/mL/cm^2^ 5 min after incubation, which significantly increased to 0.247 ± 0.032 nmol/mL/cm^2^ 60 min after incubation (*p* < 0.05). At 60 min, the amount of TTX transported at 4 °C was significantly lower (0.20 fold) than the value at 37 °C (*p* < 0.05), indicating that the urinary excretion of TTX is temperature-dependent.

Time course profiles of TTX renal reabsorption (from the apical to the basolateral side across the LLC-PK_1_ cell monolayers) are shown in Figure 1B. At an incubation temperature of 37 °C, the amount of TTX transported was 0.052 ± 0.013 nmol/mL/cm^2^ 5 min after incubation, which linearly increased to 0.483 ± 0.126 nmol/mL/cm^2^ 60 min after incubation (*p* < 0.05). At an incubation temperature of 4 °C, the amount of TTX transported was 0.012 ± 0.002 nmol/mL/cm^2^ 5 min after incubation, which increased to 0.091 ± 0.007 nmol/mL/cm^2^ 60 min after incubation (*p* < 0.05). At 60 min, the amount of TTX transported was significantly lower (0.19 fold) than the value at 37 °C (*p* < 0.05), indicating that the renal reabsorption of TTX is temperature-dependent. These results indicate that the movement of TTX is both a transcellular and carrier-mediated process. The apparent permeability coefficients of TTX (P_app_) for excretion and reabsorption are 1.95 ± 0.40 (10^−6^ cm/s) and 2.66 ± 0.66 (10^−6^ cm/s), respectively. There were no statistically significant differences between the two values (*p* > 0.05).

**Figure 2:**
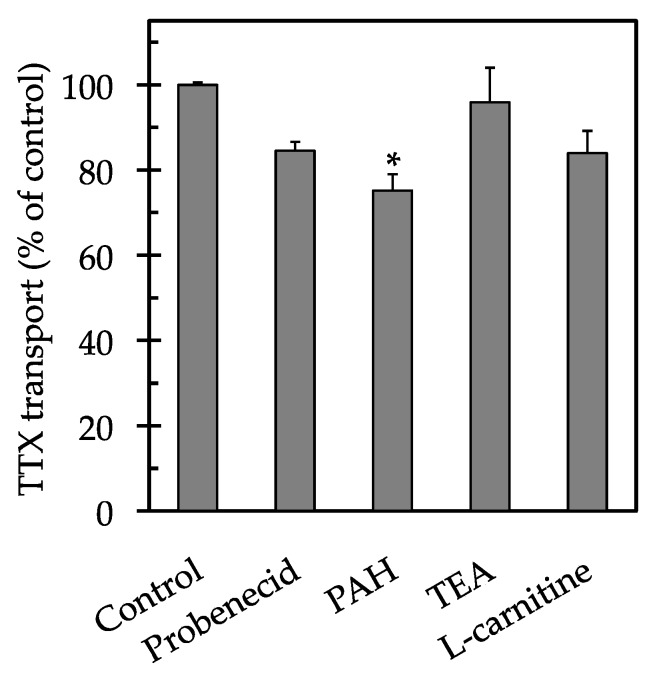
Effects of transport inhibitors on the renal reabsorption of TTX across the LLC-PK_1_ cell monolayers. The cell monolayers were incubated at 37 °C for 30 min with 50 µM TTX added to the apical side in the absence (control) or presence of 5 mM of a transport inhibitor. Each column and vertical bar represent the mean ± SE of an experiment performed in triplicate. An asterisk (*) indicates significant differences from the control value analyzed by Dunnett’s test (*p* < 0.05).

To investigate the transport characteristics of TTX across the LLC-PK_1_ cell monolayers, the effects of several transport inhibitors on the transcellular movement of TTX were examined. When the renal reabsorption of TTX was examined (Figure 2), *p*-aminohippuric acid (PAH) significantly reduced the transport of TTX, with value of 75 ± 4% compared to the control (*p* < 0.05). No significant inhibitory effects were observed after the addition of probenecid, tetraethylammonium (TEA) and l-carnitine, with values of 85 ± 2%, 96 ± 8% and 84 ± 5%, respectively, compared to the control (*p* > 0.05).

**Figure 3:**
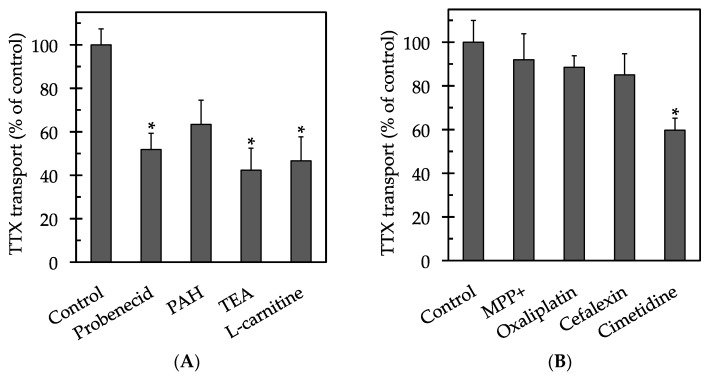
Effects of transport inhibitors on the urinary excretion of TTX across the LLC-PK_1_ cell monolayers. The cell monolayers were incubated at 37 °C for 30 min with 50 µM TTX added to the basolateral side in the absence (control) or presence of 5 mM of a transport inhibitor. Panel (**A**) shows the results of the inhibition assay used to assess multidrug resistance-associated proteins (MRPs), organic anion transporters (OATs), organic cation transporters (OCTs), and organic cation/carnitine transporters (OCTNs); panel (**B**) shows the results of the inhibition assay used to assess multidrug and toxic compound extrusion transporters (MATEs). Each column and vertical bar represent the mean ± SE of individual three experiments performed in triplicate. An asterisk (*) indicates significant differences from the control value analyzed by Dunnett’s test (*p* < 0.05).

When the urinary excretion of TTX was examined (Figure 3A), TEA, l-carnitine, and probenecid significantly reduced the movement of TTX, with values of 42 ± 10%, 47 ± 11%, and 52 ± 8%, respectively, compared to the control (*p* < 0.05). PAH moderately reduced the transport of TTX, with a value of 63 ± 11% compared to the control (*p* > 0.05). In a separate inhibition assay, cimetidine significantly reduced the transport of TTX, with a value of 60 ± 6% compared to the control (*p* < 0.05). In contrast, 1-methyl-4-phenylpyridinium (MPP+), oxaliplatin, and cefalexin had no significant effects on transport (*p* > 0.05), with values of 92 ± 12%, 88 ± 5%, and 85 ± 10%, respectively, compared to the control (Figure 3B).

## 3. Discussion

This study examined the transport characteristics of TTX excretion and reabsorption modeled using the porcine renal proximal tubule epithelial cell line LLC-PK_1_. In this study, time course profiles of TTX transport confirmed its temperature-dependent properties, indicating that TTX is transported by a carrier-mediated transcellular pathway. The temperature-dependent transcellular transport of organic cations and anions, such as ciprofloxacin, l-dopa and *N*′-methylnicotinamide, across LLC-PK_1_ cell monolayers has been also reported [23,24,25]. Saito et al. [26] revealed that the transport activity of TEA across LLC-PK_1_ cell monolayers was temperature-dependent and increased significantly during the cell culture period of 2–6 days, although the protein content of the cell monolayers did not increase appreciably during the culture. Thus, the development of TEA transport activity was not due to an increase in density of cell monolayers, but due to a cell differentiation. The TEER values of LLC-PK_1_ cell monolayers became stable from the fourth day after seeding at confluent density [27]. These findings demonstrate the validity of well-grown and differentiated LLC-PK_1_ cell monolayers as a model for the renal proximal tubular excretion and reabsorption of the organic ions.

Numerous endogenous metabolites, drugs, and xenobiotics are excreted from the bloodstream into the urine through the renal proximal tubules and/or via glomerular filtration [28]. The renal excretion of organic anions, cations, and solutes is mediated by transporter proteins such as multidrug resistance-associated protein (MRP), organic anion transporter (OAT), organic cation transporter (OCT), organic cation/carnitine transporter (OCTN), and multidrug and toxic compound extrusion transporter (MATE) [29,30,31]. In this study, the excretion of TTX was remarkably reduced by TEA and l-carnitine, which are typical substrates of OCTs and OCTNs [32]. It is known that the transport systems of organic cations are maintained in LLC-PK_1_ cells, and porcine OCT2 has been cloned from the basolateral membrane of these cells [33,34]. It appears that TTX is excreted mainly by OCTs, which are expressed in the basolateral membrane, and OCTNs, which are expressed in the apical membrane, because TTX has a pKa of 8.76 and is cationic under neutral conditions [35,36]. In the present study, the excretion of TTX was also slightly reduced by PAH (*p* > 0.05), a typical OAT substrate, and was significantly reduced by probenecid (*p* < 0.05), a typical substrate of both OATs and MRPs [37,38]. Porcine MRP1 and MRP2 are both expressed in the apical membrane of LLC-PK_1_ cells [39]. These results suggest that TTX is excreted by MRPs rather than by OATs. On the other hand, the reabsorption of TTX was significantly reduced by PAH (*p* < 0.05), while the other inhibitors had no significant effect (*p* > 0.05). These results indicate that the transporters involved in the movement of TTX differ by the direction of transport; that is, excretion vs. reabsorption.

**Figure 4:**
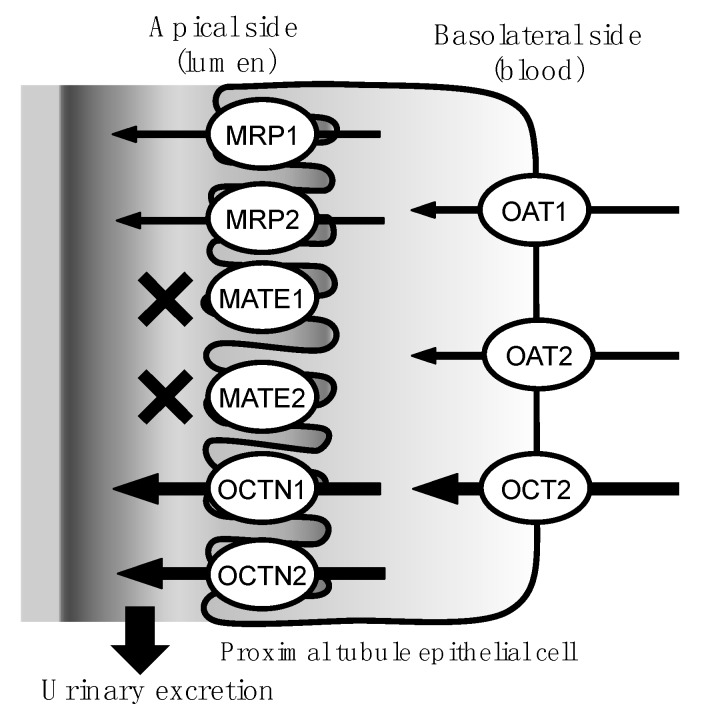
Proposed pathway of transporters involved in the urinary excretion of TTX. This study suggests that TTX is mainly transported by organic cation transporters (OCTs) and organic cation/carnitine transporters (OCTNs), and partially transported by organic anion transporters (OATs) and multidrug resistance-associated proteins (MRPs). The inhibition assay results indicate that the transport of TTX by multidrug and toxic compound extrusion transporters (MATEs) is negligible.

TEA, which is known to be transported by OCTNs and MATEs [40,41,42], most strongly reduced the transport of TTX in the present study. To investigate the involvement of MATEs in the urinary excretion of TTX, we performed another inhibition assay series with MATE inhibitors and demonstrated that MPP+, oxaliplatin, and cephalexin, typical inhibitors of MATE1 and MATE2 [42], did not affect the transport of TTX, whereas cimetidine, an inhibitor of both MATEs and OCTs, significantly reduced the transport of TTX [43,44]. These results indicate that TTX is primarily excreted by OCTs and OCTNs, partially excreted by OATs and MRPs, and negligibly excreted by MATEs, which are expressed in the apical membrane (Figure 4).

Drugs and metabolic products that bind to plasma proteins are generally not subjected to glomerular filtration. These compounds are known to be secreted into the lumen of the renal proximal tubule from the capillary blood vessel by organic ion transporters located on proximal tubular cell membranes. Indeed, we previously reported that TTX binds to both human alpha-1-acid glycoprotein and bovine serum albumin [45]. Hence, the urinary excretion of TTX is predicted to be affected by plasma protein binding and dependent on the transporters located on the proximal tubular cell membranes. Lan et al. [46] and Oh et al. [47] reported that the severity of TTX poisoning might be influenced by the presence of renal diseases, such as uremia. Nakashima et al. [48] suggested that renal dysfunction causes an accumulation of TTX due to its delayed excretion, thus changing the clinical course of TTX poisoning. Furthermore, Yu et al. [22] and Fong et al. [49] reported that the urinary concentration of TTX corrected by the urinary creatinine concentration better correlated with the severity of poisoning symptoms than did the urinary TTX concentration without the correction. These findings suggest that renal clearance (the urinary excretion rate) is intimately associated with TTX excretion, and that the promotion and/or activation of renal elimination aids early recovery from TTX poisoning. Given that renal clearance is the sum of glomerular filtration and secretion from renal proximal tubule epithelial cells, further investigation is needed to understand the TTX excretion pathway.

## 4. Materials and Methods

### 4.1. Materials

The LLC-PK_1_ cell line (ECACC number 86121112, Lot: 13G014) was purchased from DS Pharma Biomedical company (Osaka, Japan). High-glucose Dulbecco’s modified Eagle’s medium (DMEM), non-essential amino acid (NEAA) solution, and antibiotic-antimycotic solution (penicillin-streptomycin-amphotericin B suspension) were purchased from Wako Pure Chemical Industries (Osaka, Japan). Fetal bovine serum (FBS) was purchased from HyClone Laboratories (Logan, UT, USA). Crystalline TTX was purchased from Wako Pure Chemical Industries; in addition to its use in the experiments, it was used as a standard for liquid chromatography-tandem mass spectrometry (LC-MS/MS) analyses. Transport inhibitors l-carnitine, tetraethylammonium chloride (TEA), *p*-aminohippuric acid (PAH), probenecid, 1-methyl-4-phenylpyridinium iodide (MPP+), oxaliplatin, cefalexin, and cimetidine were purchased from Wako Pure Chemical Industries. All other chemicals were of reagent-grade quality.

### 4.2. Cell Culture

LLC-PK_1_ cells were cultured in a 75-cm^2^ flask (Thermo Fisher Scientific, Waltham, MA, USA) using high-glucose DMEM supplemented with 10% FBS, 1% NEAA, 100 U/mL penicillin, 100 µg/mL streptomycin, and 0.25 µg/mL amphotericin B. The cells were incubated at 37 °C in a humidified atmosphere containing 5% CO_2_. The medium was changed every two days. The confluent cells were subcultured weekly by trypsinization with a solution of 0.25% trypsin containing 1 mM EDTA [50,51] and seeded at a density of 4 × 10^4^ cells/cm^2^. For the transport experiments, the cells were seeded at a density of 6 × 10^5^ cells/cm^2^ in 24-well Transwell^®^ inserts containing a polyester membrane (pore size 1.0 µm, 0.3 cm^2^ growth surface area; BD Falcon, Franklin Lakes, NJ, USA). Transwell inserts were placed in a 24-well companion plate (BD Falcon), and 0.3 mL and 1 mL of the medium were added to apical and basolateral sides of the Transwell insert chambers, respectively. The medium was changed on 4 days after seeding, and the seeded cells were maintained for 7 days to prepare differentiated cell monolayers.

### 4.3. Transepithelial Electrical Resistance (TEER) Measurements

TEER was measured using a Millicell ERS-2 voltohmmeter (Merck Millipore, Billerica, MA, USA). The ohmic resistance of the LLC-PK_1_ cell monolayers (R_tissue_) was normalized by subtracting the background of the blank filter (R_blank_) from the sample (R_total_). TEER values of the cell monolayers (TEER_tissue_) (Ω·cm^2^) were calculated from the following equations:

R_tissue_ (Ω) = R_total_ (Ω) − R_blank_ (Ω) (1)

TEER_tissue_ = R_tissue_ (Ω)·S (cm^2^) (2)

where S is the surface area of the LLC-PK_1_ cell monolayers (cm^2^). The cell monolayers, whose TEER values were above 20 Ω·cm^2^, were used for the experiments with reference to TEER values reported previously [25,26,52,53].

### 4.4. Transport Studies

Transcellular transport of TTX was examined using LLC-PK_1_ cell monolayers grown in Transwell inserts on day 7 after seeding. The medium was aspirated from both chambers separated by the cell monolayers, and the cell monolayers were washed twice with transport buffer (137 mM NaCl, 5.4 mM KCl, 0.44 mM Na_2_HPO_4_, 0.39 mM KH_2_PO_4_, 0.95 mM CaCl_2_, 0.81 mM MgSO_4_, 10 mM HEPES, and 10 mM d-glucose, adjusted to pH 7.4 with NaOH). The cell monolayers were preincubated at 37 °C for 10 min. The time course study of transcellular TTX transport was begun by replacing the buffer in the apical or basolateral side chamber (donor) with transport buffer (pH 7.4) containing 50 µM TTX. Incubation was carried out at 37 °C for up to 60 min. At the designated times, the incubation buffer in the opposite-side chamber (receiver) was rapidly collected. Additionally, the incubation temperature was lowered to 4 °C to examine the temperature-dependence of the transport.

The inhibitory effects of the transporter inhibitors on the movement of TTX across the cell monolayers were investigated. The inhibitors (probenecid, PAH, TEA, l-carnitine, MPP+, oxaliplatin, cefalexin, and cimetidine) were dissolved in transport buffer (pH 7.4) containing 50 µM TTX and 1% methanol to final concentrations of 5 mM. After the preincubation described above, the cell monolayers were incubated at 37 °C for 30 min with transport buffer (pH 7.4) containing 50 µM TTX and 5 mM of each inhibitor in the donor chamber (basolateral or apical side). The transport buffer (pH 7.4) in the receiver chamber was rapidly collected at the end of the incubation period and subjected to LC-MS/MS for determination of TTX levels.

### 4.5. Determination of TTX Levels by LC-MS/MS

Sample solutions were diluted five times with MilliQ water to mitigate the effects of background noise from by the salts in the incubation buffer and filtered through disposable syringe filter units (DISMIC-25CS, Advantec Toyo Kaisha, Tokyo, Japan). TTX quantification was performed by LC-MS/MS analysis according to the method of Matsumoto et al. [54]. The LC-MS/MS system consisted of an API 2000 triple-quadrupole tandem mass spectrometer (Sciex, Framingham, MA, USA) and an Agilent 1100 Infinity HPLC system (Agilent Technologies, Santa Clara, CA, USA). The analytical column was a TSKgel Amide-80 column (2.0 i.d. × 150 mm, 3 µm particle size; Tosoh, Tokyo, Japan) that was maintained at 35 °C. Mobile phases A and B consisted of 16 mM ammonium formate (pH 5.5) and acetonitrile, respectively, and the flow rate was set at 200 µL/min. The elution profile was 55% B (0–10 min), 90% B (10–13 min), and 55% B (13–20 min). The injection volume was 10 µL. The eluate was directly induced into the ion-source block of the LC-MS/MS system. TTX was ionized in positive-ion mode, and the fragment ion at (m/z) 162 that results from the dissociation of the parent ion of TTX at (m/z) 320 was detected in multiple reaction-monitoring mode. The standard curves for TTX were prepared by the addition of 0.1–10 ng/mL TTX (final concentration) to the five-fold-diluted transport buffer solutions.

### 4.6. Pharmacokinetics and Statistical Analyses

The permeation rate of TTX (J) (nmol/min) was defined as the slope of the initial linear portion of the curve that was calculated by linear regression analysis from the scatter plots of the amount of TTX transported (nmol) versus time (min). The apparent permeability coefficient of TTX (P_app_) (cm/s) was calculated from the following equation:

J = P_app_·S·C (3)

where S and C are the surface area of the LLC-PK_1_ cell monolayers (cm^2^) and the initial TTX concentration in the donor chamber (µM), respectively. Data are expressed as the mean ± standard error. Dunnett’s test and Student’s *t*-test were used to analyze the significance of differences among the means at the 5% significance level.

## References

[1] Narahashi T., Moore J.W., Scott W.R. (1964). Tetrodotoxin blockage of sodium conductance increase in lobster giant axons. J. Gen. Physiol..

[2] Takata M., Moore J.W., Kao C.Y., Fuhrman F.A. (1966). Blockage of sodium conductance increase in lobster giant axon by tarichatoxin (tetrodotoxin). J. Gen. Physiol..

[3] Miyazawa K., Noguchi T. (2001). Distribution and origin of tetrodotoxin. J. Toxicol. Toxin Rev..

[4] Habu J., Kim M., Katayama M., Komiya H. (2001). Jomon subsistence-settlement systems at the Sannai Maruyama site. Bull. Indo-Pac. Prehist. Assoc..

[5] Ishida Y., Yamada A., Adachi H., Yagisawa I., Tadokoro K., Geiger H.J. (2009). Salmon distribution in the northern Japan during the Jomon Period, 2000–8000 years ago, and its implications for future global warming. NPAFC Bull..

[6] O’Connor S., Ono R., Clarkson C. (2011). Pelagic fishing at 42,000 years before the present and the maritime skills of modern humans. Science.

[7] Isbister G.K., Son J., Wang F., Maclean C.J., Lin C.S., Ujma J., Balit C.R., Smith B., Milder D.G., Kiernan M.C. (2002). Puffer fish poisoning: A potentially life-threatening condition. Med. J. Aust..

[8] Kanchanapongkul J. (2008). Tetrodotoxin poisoning following ingestion of the toxic eggs of the horseshoe crab *Carcinoscorpius rotundicauda*, a case series from 1994 through 2006. Southeast Asian J. Trop. Med. Public Health.

[9] Fernández-Ortega J.F., Morales-de los Santos J.M., Herrera-Gutiérrez M.E., Fernández-Sánchez V., Loureo P.R., Rancaño A.A., Téllez-Andrade A. (2010). Seafood intoxication by tetrodotoxin: First case in Europe. J. Emerg. Med..

[10] Islam Q.T., Razzak M.A., Islam M.A., Bari M.I., Basher A., Chowdhury F.R., Sayeduzzaman A.B., Ahasan H.A., Faiz M.A., Arakawa O. (2011). Puffer fish poisoning in Bangladesh: Clinical and toxicological results from large outbreaks in 2008. Trans. R. Soc. Trop. Med. Hyg..

[11] Yong Y.S., Quek L.S., Lim E.K., Ngo A. (2013). A case report of puffer fish poisoning in Singapore. Case Rep. Med..

[12] Simões E.M.D.S., Mendes T.M., Adão A., Junior V.H. (2014). Poisoning after ingestion of pufferfish in Brazil: Report of 11 cases. J. Venom. Anim. Toxins Incl. Trop. Dis..

[13] Wu Y.J., Lin C.L., Chen C.H., Hsieh C.H., Jen H.C., Jian S.J., Hwang D.F. (2014). Toxin and species identification of toxic octopus implicated into food poisoning in Taiwan. Toxicon.

[14] Cole J.B., Heegaard W.G., Deeds J.R., McGrath S.C., Handy S.M. (2015). Tetrodotoxin poisoning outbreak from imported dried puffer fish-Minneapolis, Minnesota, 2014. MMWR Morb. Mortal. Wkly. Rep..

[15] You J., Yue Y., Xing F., Xia W., Lai S., Zhang F. (2015). Tetrodotoxin poisoning caused by Goby fish consumption in southeast China: A retrospective case series analysis. Clinics.

[16] Noguchi T., Ebesu J.S.M. (2001). Puffer poisoning: Epidemiology and treatment. J. Toxicol. Toxin Rev..

[17] Isbister G.K., Kiernan M.C. (2005). Neurotoxic marine poisoning. Lancet Neurol..

[18] Hwang D.F., Noguchi T. (2007). Tetrodotoxin poisoning. Adv. Food Nutr. Res..

[19] Oda K., Araki K., Totoki T., Shibasaki H. (1989). Nerve conduction study of human tetrodotoxication. Neurology.

[20] O’Leary M.A., Schneider J.J., Isbister G.K. (2004). Use of high performance liquid chromatography to measure tetrodotoxin in serum and urine of poisoned patients. Toxicon.

[21] Hwang P.A., Tsai Y.H., Deng J.F., Cheng C.A., Ho P.H., Hwang D.F. (2005). Identification of tetrodotoxin in a marine gastropod (*Nassarius glans*) responsible for human morbidity and mortality in Taiwan. J. Food Prot..

[22] Yu C.H., Yu C.F., Tam S., Yu P.H. (2010). Rapid screening of tetrodotoxin in urine and plasma of patients with puffer fish poisoning by HPLC with creatinine correction. Food Addit. Contam. Part A Chem. Anal. Control Expo. Risk Assess..

[23] Sasaya M., Oda M., Endo T., Saitoh H., Takada M. (1997). The transport of ciprofloxacin in cultured kidney epithelial cells LLC-PK_1_. Biol. Pharm. Bull..

[24] Soares-Da-Silva P., Serrão M.P. (2000). Molecular modulation of inward and outward apical transporters of l-dopa in LLC-PK_1_ cells. Am. J. Physiol. Ren. Physiol..

[25] Fouda A.K., Fauth C., Roch-Ramel F. (1990). Transport of organic cations by kidney epithelial cell line LLC-PK_1_. J. Pharmacol. Exp. Ther..

[26] Saito H., Yamamoto M., Inui K., Hori R. (1992). Transcellular transport of organic cation across monolayers of kidney epithelial cell line LLC-PK_1_. Am. J. Physiol..

[27] Saladik D.T., Soler A.P., Lewis S.A., Mullin J.M. (1995). Cell division does not increase transepithelial permeability of LLC-PK_1_ cell sheets. Exp. Cell Res..

[28] Tsuji A. (2006). Impact of transporter-mediated drug absorption, distribution, elimination and drug interactions in antimicrobial chemotherapy. J. Infect. Chemother..

[29] Burckhardt G., Wolff N.A. (2000). Structure of renal organic anion and cation transporters. Am. J. Physiol. Ren. Physiol..

[30] Motohashi H., Nakao Y., Masuda S., Katsura T., Kamba T., Ogawa O., Inui K. (2013). Precise comparison of protein localization among OCT, OAT, and MATE in human kidney. J. Pharm. Sci..

[31] Yin J., Wang J. (2016). Renal drug transporters and their significance in drug-drug interactions. Acta Pharm. Sin. B.

[32] Inui K., Masuda S., Saito H. (2000). Cellular and molecular aspects of drug transport in the kidney. Kidney Int..

[33] Tomita Y., Otsuki Y., Hashimoto Y., Inui K. (1997). Kinetic analysis of tetraethylammonium transport in the kidney epithelial cell line, LLC-PK_1_. Pharm. Res..

[34] Gründemann D., Babin-Ebell J., Martel F., Ording N., Schmidt A., Schömig E. (1997). Primary structure and functional expression of the apical organic cation transporter from kidney epithelial LLC-PK_1_ cells. J. Biol. Chem..

[35] Goto T., Kishi Y., Takahashi S., Hirata Y. (1965). Tetrodotoxin. Tetrahedron.

[36] Kungsuwan A., Nagashima Y., Noguchi T., Shida Y., Suvapeepan S., Suwansakornkul P., Hashimoto K. (1987). Tetrodotoxin in the horseshoe crab *Carcinoscorpius rotundicauda* inhabiting Thailand. Nippon Suisan Gakkaishi.

[37] Feller N., Broxterman H.J., Währer D.C., Pinedo H.M. (1995). ATP-dependent efflux of calcein by the multidrug resistance protein (MRP): No inhibition by intracellular glutathione depletion. FEBS Lett..

[38] Evers R., Zaman G.J., van Deemter L., Jansen H., Calafat J., Oomen L.C., Oude-Elferink R.P., Borst P., Schinkel A.H. (1996). Basolateral localization and export activity of the human multidrug resistance-associated protein in polarized pig kidney cells. J. Clin. Investig..

[39] Goh L.B., Spears K.J., Yao D., Ayrton A., Morgan P., Roland W.C., Friedberg T. (2002). Endogenous drug transporters in vitro and in vivo models for the prediction of drug disposition in man. Biochem. Pharmacol..

[40] Tamai I., Ohashi R., Nezu J., Yabuuchi H., Oku A., Shimane M., Sai Y., Tsuji A. (1998). Molecular and functional identification of sodium ion-dependent, high affinity human carnitine transporter OCTN2. J. Biol. Chem..

[41] Yabuuchi H., Tamai I., Nezu J., Sakamoto K., Oku A., Shimane M., Sai Y., Tsuji A. (1999). Novel membrane transporter OCTN1 mediates multispecific, bidirectional, and pH-dependent transport of organic cations. J. Pharmacol. Exp. Ther..

[42] Tanihara Y., Masuda S., Sato T., Katsura T., Ogawa O., Inui K. (2007). Substrate specificity of MATE1 and MATE2-K, human multidrug and toxin extrusions/H^+^-organic cation antiporters. Biochem. Pharmacol..

[43] Otsuka M., Matsumoto T., Morimoto R., Arioka S., Omote H., Moriyama Y. (2005). A human transporter protein that mediates the final excretion step for toxic organic cations. Proc. Natl. Acad. Sci. USA.

[44] Tahara H., Kusuhara H., Endou H., Koepsell H., Imaoka T., Fuse E., Sugiyama Y. (2005). A species difference in the transport activities of H_2_ receptor antagonists by rat and human renal organic anion and cation transporters. J. Pharmacol. Exp. Ther..

[45] Matsumoto T., Tanuma D., Tsutsumi K., Jeon J.K., Ishizaki S., Nagashima Y. (2010). Plasma protein binding of tetrodotoxin in the marine puffer fish *Takifugu rubripes*. Toxicon.

[46] Lan M.Y., Lai S.L., Chen S.S., Hwang D.F. (1999). Tetrodotoxin intoxication in a uraemic patient. J. Neurol. Neurosurg. Psychiatry.

[47] Oh J., Sunwoo M.K., Sunwoo I.N. (2011). Serial electrophysiological changes in uraemic patients with tetrodotoxin intoxication. Clin. Neurophysiol..

[48] Nakashima R., Nakata Y., Kameoka M., Hayashi N., Watanabe K., Yagi K. (2007). A case of tetrodotoxin intoxication in a uremic patient. Chudoku Kenkyu.

[49] Fong B.M., Tam S., Tsui S.H., Leung K.S. (2011). Development and validation of a high-throughput double solid phase extraction-liquid chromatography-tandem mass spectrometry method for the determination of tetrodotoxin in human urine and plasma. Talanta.

[50] Inui K., Saito H., Hori R. (1985). H^+^-gradient-dependent active transport of tetraethylammonium cation in apical-membrane vesicles isolated from kidney epithelial cell line LLC-PK_1_. Biochem. J..

[51] Endo T., Kimura O., Sasaya M., Takada M., Sakata M. (1995). Na^+^- and energy-dependent transport of cadmium into LLC-PK_1_ cells. Biol. Pharm. Bull..

[52] Masago M., Takaai M., Sakata J., Horie A., Ito T., Ishida K., Taguchi M., Hashimoto Y. (2010). Membrane transport mechanisms of quinidine and procainamide in renal LLC-PK_1_ and intestinal LS180 cells. Biol. Pharm. Bull..

[53] Takaai M., Suzuki H., Ishida K., Tahara K., Hashimoto Y. (2007). Pharmacokinetic analysis of transcellular transport of levofloxacin across LLC-PK_1_ and Caco-2 cell monolayers. Biol. Pharm. Bull..

[54] Matsumoto T., Kiriake A., Ishizaki S., Watabe S., Nagashima Y. (2015). Biliary excretion of tetrodotoxin in the cultured pufferfish *Takifugu rubripes* juvenile after intramuscular administration. Toxicon.

